# Physiological and psychological stress reactivity in narcolepsy type 1

**DOI:** 10.1093/sleep/zsae265

**Published:** 2024-11-15

**Authors:** Marieke Vringer, Denise Bijlenga, Jingru Zhou, Onno C Meijer, Christiaan H Vinkers, Gert Jan Lammers, Rolf Fronczek

**Affiliations:** Stichting Epilepsie Instellingen Nederland (SEIN), Sleep-Wake Center, Heemstede, The Netherlands; Department of Neurology, Leiden University Medical Center (LUMC), Leiden, The Netherlands; Stichting Epilepsie Instellingen Nederland (SEIN), Sleep-Wake Center, Heemstede, The Netherlands; Department of Neurology, Leiden University Medical Center (LUMC), Leiden, The Netherlands; Stichting Epilepsie Instellingen Nederland (SEIN), Sleep-Wake Center, Heemstede, The Netherlands; Department of Neurology, Leiden University Medical Center (LUMC), Leiden, The Netherlands; Department of Neuroendocrinology, Leiden University Medical Center (LUMC), Leiden, The Netherlands; Department of Psychiatry and Department of Anatomy & Neurosciences, Amsterdam University Medical Center (AUMC), Amsterdam, The Netherlands; Amsterdam Neuroscience, Mood, Anxiety, Psychosis, Stress & Sleep Program, Amsterdam, The Netherlands; Amsterdam Public Health, Mental Health Program, Amsterdam, The Netherlands; GGZ InGeest, Academic Working Place Depression, Amsterdam, The Netherlands; Stichting Epilepsie Instellingen Nederland (SEIN), Sleep-Wake Center, Heemstede, The Netherlands; Department of Neurology, Leiden University Medical Center (LUMC), Leiden, The Netherlands; Stichting Epilepsie Instellingen Nederland (SEIN), Sleep-Wake Center, Heemstede, The Netherlands; Department of Neurology, Leiden University Medical Center (LUMC), Leiden, The Netherlands

**Keywords:** corticotrophin-releasing hormone, trier social stress test for groups, adrenocorticotropic hormone, cortisol, HPA-axis

## Abstract

**Study Objectives:**

Narcolepsy type 1 (NT1) is a chronic sleep–wake disorder, characterized by a loss of hypocretin production. Unexpectedly, in postmortem tissue of people with NT1, there is a loss of corticotrophin-releasing hormone (CRH) in the paraventricular nucleus. CRH is known as an activator of the hypothalamic-pituitary-adrenal axis in response to stress. This activation results in the release of the stress hormones adrenocorticotropic hormone (ACTH) and cortisol. We hypothesize an altered physiological and psychological stress response in NT1.

**Methods:**

Participants were people with NT1 (*n* = 14) and matched healthy controls (*n* = 12). The Trier Social Stress Test for Groups (TSST-G), a validated socially evaluated stress test in controlled settings, induced acute stress. We measured ACTH and cortisol levels in the blood before and at three timepoints after the TSST-G. We also measured subjective stress and heart rate levels.

**Results:**

In both groups, acute stress led to increases in ACTH (*p* = .006), cortisol (*p* < .001), heart rate (*p* < .001), and subjective stress (*p* < .001). Subjectively, people with NT1 experienced more stress than controls (*p* < .001). No differences were found in heart rate, cortisol, and ACTH between people with NT1 and controls at any timepoint. Secondary analyses showed that men with NT1 had lower cortisol levels immediately after stress induction than men in the control group (*p* = .002).

**Conclusions:**

People with NT1 show an increased subjective stress response, but no changes in their endocrine or cardiovascular stress reactivity. Further research is required to determine the impact of reduced CRH production and gender in NT1.

## Graphical Abstract

**Graphical Abstract:**
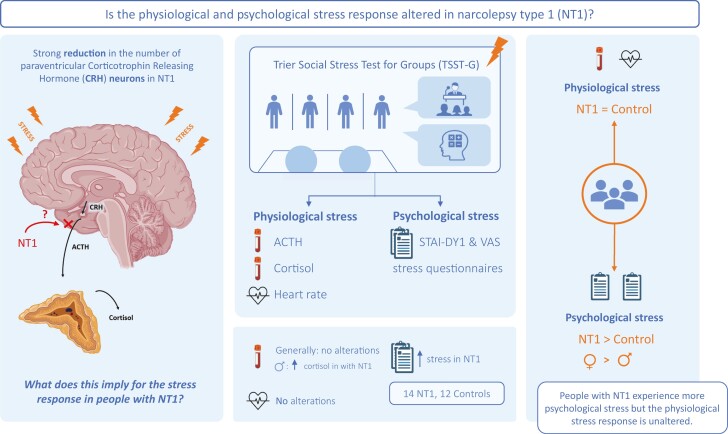

**Statement of Significance**

Narcolepsy type 1 (NT1) is a chronic sleep–wake disorder presumed to be caused by auto-immune processing targeting hypocretin-producing neurons. Unexpectedly, also reduced corticotrophin-releasing hormone (CRH) production was described in the paraventricular nucleus of people with NT1. CRH from this brain region is responsible for the release of stress hormones after acute stress. We therefore investigated the impact of acute stress on various physiological and psychological stress markers in individuals with NT1 and controls. We show that people with NT1 experienced more stress while, generally, no changes in their physiological stress reactivity were observed. A possible gender effect cannot be ruled out since men with NT1 may have a lowered cortisol stress response compared to control men.

Narcolepsy type 1 (NT1) is a rare and disabling sleep–wake disorder. It is characterized by cataplexy (episodes with loss of muscle tone triggered by mostly positive emotions) and sleep–wake dysregulation including excessive daytime sleepiness, sleep paralysis, hypnagogic hallucinations, and disturbed nocturnal sleep [1]. Deficiencies in the hypocretin (Hcrt, also known as orexin) system most likely cause these symptoms [1–5]. Besides the loss of Hcrt-positive neurons in postmortem tissue of people with NT1, an 88% loss of corticotrophin-releasing hormone (CRH)-positive neurons in the paraventricular nucleus (PVN) of people with NT1 has been reported [3]. CRH-positive neurons in the thalamus and locus coeruleus were however unaffected [3]. Hcrt and CRH neurons may be destroyed in NT1 due to an auto-immune process, or be less active due to genetic silencing [3, 6, 7].

CRH in the PVN is related to stress and activation of the sympathetic nervous system and is known for its role as a hypothalamic-pituitary-adrenal (HPA)-axis activator [8, 9]. Within the HPA-axis, CRH is released by the PVN of the hypothalamus as a reaction to acute stress [9, 10]. The released CRH is transported by blood to the anterior pituitary gland, where it stimulates adrenocorticotropic hormone (ACTH) release. In turn, ACTH activates cortisol synthesis and secretion by the adrenal cortex [11, 12]. In rodents, a functionally distinct subpopulation of CRH neurons in the PVN was identified; these neurons have pre-autonomic projections to the sympathetic preganglionic neurons in the spinal cord [13]. This subpopulation may affect sympathetic mechanisms in the stress response, such as an increased heart rate.

Previous research in rodents showed the effect of impaired CRH signaling on the HPA axis. CRH is considered the predominant stimulator of ACTH release in the pituitary gland [14], with co-released vasopressin as a potentiating factor. Mice with CRH loss in the hypothalamus, but with intact CRH expression in the amygdala and cerebral cortex, showed decreased basal plasma ACTH and decreased corticosterone secretion, which is the equivalent of cortisol in humans [15]. Specific silencing of CRH expression in the PVN using RNA interference decreased stress-induced levels of ACTH in mice [16]. Centrally administered Hcrt-1 peptide in rats leads to increased plasma levels of ACTH and corticosterone [17, 18]. These results align with the previously observed decreased basal ACTH secretion in people with NT1 in comparison to healthy individuals, but not to unchanged basal cortisol levels [19]. Additionally, HPA dysregulation has been associated with anxiety and depression symptoms [20, 21], which are also commonly observed in people with NT1 [22, 23].

Loss of CRH in the PVN of people with NT1 might result in dysfunctional HPA-axis activation. This would be reflected in a lowered ACTH and cortisol response to acute stress. People with NT1 may also have a reduced heart rate stress response due to the loss of pre-autonomic CRH subpopulations. For this reason, we investigated various physiological (endocrine and cardiovascular) stress markers and psychological (subjectively reported) stress before and after a stress test. The role of gender is also analyzed as a secondary outcome. A better understanding of the response to acute stress in people with NT1 will give better insights into the effects of the lost CRH signal in the PVN, possibly related to depression and anxiety symptoms in people with NT1.

## Methods

### Participants

We included people with NT1 and healthy controls, matched for sex and age (maximum of 7 years difference). As this study was designed as a proof-of-principle, the sample size was based on previous research where a 40%–60% reduction of stress-induced ACTH was observed in animals with PVN-specific CRH inhibition [16], and on the 88% decrease of CRH-positive cells that were found in the PVN of people with NT1 [3]. Combined with the average increase of cortisol levels after the stress induction of about 6.5 µg/dl (SD = 2.5) [24], we aimed to include 12 participants per group. All people with NT1 were recruited from the sleep–wake center of SEIN in Heemstede, the Netherlands, which is a specialized referral center for central hypersomnolence disorders. Healthy controls were recruited through online advertising.

Participants in the NT1 group were between 18 and 64 years old and formally diagnosed with NT1 following ICSD-3 criteria. Exclusion criteria were: current depressive disorder, learning disability, any disease that may affect HPA-activity, untreated hypertension, use of benzodiazepines or any hormonal treatment (other than oral contraceptives), smoking >5 cigarettes a day, excessive alcohol consumption (women >14 and men >21 alcohol units/week), pregnancy or lactating, no fluency in the Dutch language, speech impairments, being an experienced public speaker or mental health professional, or any specialization in psychology. Additionally, people with (a history of) anxiety disorder, panic attacks, cardiac disease, epilepsy, or Cushing’s disease were not eligible. Additional exclusion criteria for the controls were any sleep–wake disorder or use of any sleep medication or antidepressants. People with NT1 using psycho-stimulants (e.g. methylphenidate, modafinil) were asked to discontinue these medications on the test day. Other narcolepsy-specific medications were continued.

Participants were unaware of the stress-inducing procedure during the experiment as they had been informed beforehand that the study aimed to examine social resilience. This was needed to avoid participation bias regarding stress coping strategies. Before participation, all participants provided informed consent. The study was approved by the ethics committee of Leiden-Den Haag-Delft (protocol #NL82071.058.22).

### Procedure

**Figure 1.**
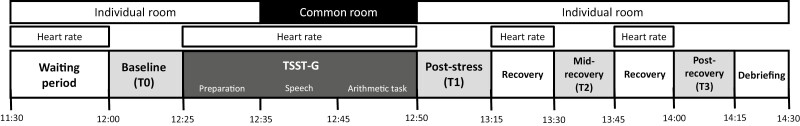
Schematic overview of a study day. TSST-G, trier social stress test for groups.

Each participant had one test day, lasting from 11:30 to 14:30. See Figure 1 for an overview of the test day procedure. During the test day, a strict schedule was applied to minimize the influence of circadian fluctuation in cortisol and ACTH levels [25]. There were seven test days in total, each involving two to four participants, and where people with NT1 and controls were represented. We ensured the participants did not know each other on a given test day. One hour before the baseline assessment, participants had no meals, drinks (other than water), or exercise. Each participant had an individual room.

After arrival on the test day, participants were asked to wear an electrocardiogram (ECG) vest with an attached heart rate wearable device (Nuubo®, Madrid, Spain). Gel (Tensive®) was added to the electrodes in the vest to increase skin conductance. An intravenous cannula was inserted for blood sampling during the test day. Participants were then also asked to fill out a general questionnaire about their demographics, sleep duration the night before the test, current use of medication (including contraceptives), and age of NT1 symptom onset (for people with NT1). Participants completed the Epworth Sleepiness Scale (ESS) and the Fatigue Severity Scale (FSS). The ESS has eight items with a range of 0–24. An ESS score >10 indicates excessive daytime sleepiness. The FSS has nine items with a range of 1–7, where a score ≥4 implies moderate to severe fatigue.

After these preparations, the testing started with a 30-minute waiting period in their respective rooms, where emotionally neutral reading material and puzzles were available (Figure 1). This waiting period was applied so that participants could acclimatize. The subsequent baseline (T0) assessment was conducted while participants were at rest. After the T0 assessment, the participants were asked to prepare a speech for the first part (speech preparation) of the Trier Social Stress Test for Groups (TSST-G; see paragraph 2.5). Participants were taken to the common room for the second and third parts (speech and arithmetic tasks) of the TSST-G. They were placed next to other participants, separated by screens, hence avoiding non-verbal interaction between participants. After the TSST-G, the participants were directed to their rooms for the post-stress (T1) assessment. The mid-recovery (T2) assessment was after a 15-minute recovery period, and the post-recovery (T3) assessment was after another 15-minute recovery period. After T3, participants were informed about the true nature of the study and had the opportunity to ask questions during an extended debriefing.

### Physiological stress measurements

#### Endocrine stress

Blood samples were taken to measure ACTH and cortisol as physiological stress markers. At each timepoint, 4 mL blood was drawn in an Aprotinin-EDTA plasma tube (VACUETTE® PREMIUM) and a 3.5 mL SST serum tube (BD Vacutainer® SST II Plus). Blood was immediately centrifuged at 4000 rpm for 10 minutes. The plasma and serum were stored at -80°C before processing at the Clinical Chemistry and Laboratory Medicine at the Leiden University Medical Center. An electrochemiluminescence immunoassay (ECLIA) was used to determine ACTH and cortisol levels.

#### Cardiovascular stress

Heart rate was continuously recorded several times on the test day using the Nuubo® vest containing multiple electrodes. The ECG data were imported in Python and the Leonardo software (Nuubo®) to determine the mean beats per minute (bpm) for each recording.

### Psychological stress measurements

At all timepoints, momentary subjective psychological stress was assessed using the first part of the Dutch version of the State and Trait Anxiety Inventory (STAI-DY1), which is a validated and widely used questionnaire, and also often used to measure the psychological response to stress in TSST(-G) experiments [26]. Additionally, participants rated their level of perceived psychological stress, anxiety, insecurity, perspiration (previously used in [27] and [28]), energy, and sleepiness levels on a set of six self-constructed horizontal 100 mm visual analog scales (VAS), ranging from “not at all” to “extremely,” by placing an “x” on the horizontal line. The main psychological stress outcomes were the STAI-DY1 and the VAS “stress” scores (further referred to as “VAS stress”).

### Trier social stress test for groups (TSST-G)

The TSST-G is a widely used method in social science to induce social stress in experiments. It was developed by von Dawans et al. (2011) as a standardized stress test performed in small groups where it is possible to test multiple participants simultaneously [26] and consists of three main phases: (1) speech preparation, (2) giving a free speech, and (3) a mental arithmetic task (Figure 1).

#### Phase 1: Speech preparation

Participants received written instructions stating they had 10 minutes to prepare a two-minute speech for a job application. The speech would be presented to a panel trained to observe non-verbal communication with additional voice and behavior analyses. They were told that the panel would provide an additional task after their speech performance. Participants prepared their speeches in their respective individual rooms; they could write down notes during the preparation but could not use the notes during the speech.

**Figure 2.**
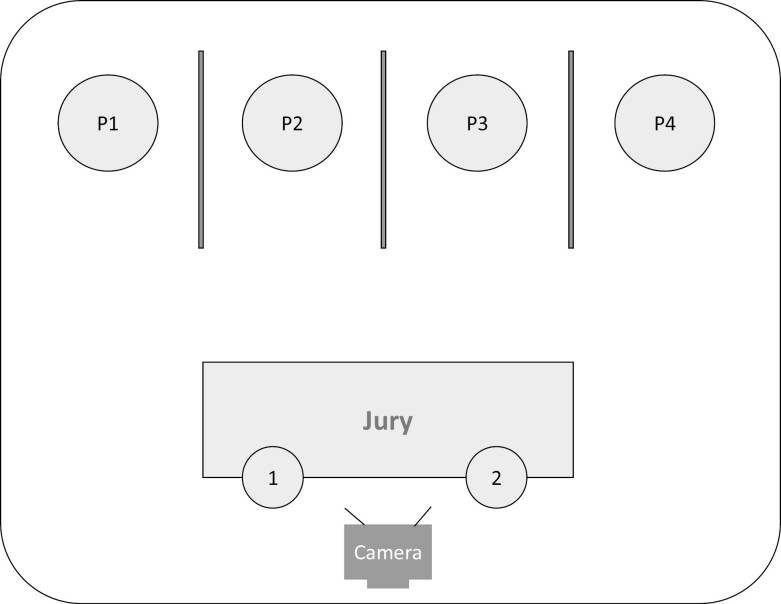
Setup of the common room. The speech and arithmetic tasks of the Trier Social Stress Test for Groups were performed in front of two judges. Participants were placed between screens to avoid non-verbal interaction between participants.

The speech and arithmetic tasks were performed before the panel in the common room [29, 30]. The participants were placed in front of two panelists and a (non-recording) camera (Figure 2). Participants could hear the performance of the others. The two panelists were trained for their task and instructed to keep a neutral face during the whole TSST-G, and not to respond to the participants in a socially accepted way (e.g. nodding or using affirmative vocalization).

#### Phase 2: Public speech

The participants were asked to perform their speech in a random order. Each speech was given in two parts of one minute to maintain increased stress levels. The panel did not interrupt when participants were talking, and in case of silence for more than 10 seconds, a panel member would interject by saying “you still have time, please continue.” If a participant again did not talk for a few seconds, the panel asked a prepared negatively stated question, e.g. *“*what are your worst personality traits?” The total duration of the speeches was up to 8 minutes.

#### Phase 3: Arithmetic task

After the speeches, the panel introduced the arithmetic task. Participants were asked to serially subtract 17 from a given 4-digit number as quickly and accurately as possible. If a mistake was made, the panel interjected with “no,” followed by the correct answer, and instructed the participant to start over. Participants performed this task in a random order in two parts of 45 seconds, beginning with a different number for each participant to avoid learning effects. The total duration of this phase was up to six minutes.

### Statistical analysis

Analyses of variance (ANOVAs) were used to test for group differences between the NT1 group and controls in age, gender, body mass index (BMI), and scores on the FSS and ESS. ANOVAs were also used to test whether variances in the baseline physiological stress markers ACTH, cortisol, and heart rate could be explained by the use of stimulant medication, a different TSST-G session, or the use of oral contraceptives.

A repeated measures linear mixed model (LMM) was conducted to test for differences between people with and without NT1 in ACTH and cortisol levels, heart rate, and scores on the VAS stress and STAI-DY1 questionnaires over the timepoints. A log transformation was applied to all six components of the VAS and STAI-DY1 scores to increase normality. Within the model, the subject was included as a random effect. Timepoint and Group (NT1 or control) were included as fixed effects. Group × Timepoint was included as an interaction term. A secondary multivariate LMM was conducted to test for the effect of gender. In the multivariate model, Gender was added as the fixed effect and Gender × Group was included as the interaction effect. A second LMM without an interaction term was conducted when no interaction effect was found. If there was an interaction effect of Gender × Group, an ANOVA was performed at each timepoint for both sexes separately to find out how and when gender affected the group difference.

Individual areas under the curves (AUC) of ACTH, cortisol, heart rate, VAS stress, and STAI-DY1 values were established. By calculating the AUC, the total effect of the stress outcome measures can be assessed over all timepoints, rather than at individual timepoints only. The AUC calculations were based on the absolute ground values (AUCg) without correction for baseline, and the increased values (AUCi), where the values were corrected for baseline [31]. The mean group AUCg and AUCi were compared between the NT1 and control groups using ANOVAs.

Pearson’s correlations were used between each of the individual stress outcome measurements and between the duration of NT1 symptoms and each baseline stress outcome.

We used IBM SPSS® version 29 for the statistical analyses. The significance level was set to α = 0.05 (two-sided).

## Results

### Participants

Thirty-five people were screened for eligibility during a telephone assessment, of whom 26 (16 women and 10 men) were included (Table 1). Two age-matched pairs had an age difference of 5 and 6 years, all other matched pairs differed by 0–3 years in age. There were no differences in age, gender, and BMI between the NT1 and control groups. As expected, participants with NT1 scored higher on the FSS (*p* < .001) and the ESS (*p* < .001) questionnaires. See Table S1 for information about current prescribed medication use and its indication per participant.

**Table 1.** General Characteristics

	NT1, *n* = 14	Controls, *n* = 12	One-way ANOVA outcomes, *P*-value
Women, *n* (%)	8 (57.14%)	8 (66.67%)	F = 0.231, *p* = .635
Age in years, mean (SD)	33.50 (12.35)	31.58 (10.68)	F = 0.176, *p* = .679
Range	21-55	19-49	
BMI^1^, mean (SD)	24.26 (4.56)	25.07 (4.19)	F = 0.217, *p* = .645
FSS^2^, mean (SD)	38.57 (10.69)	22.58 (6.08)	F = 20.948, *p* < .001^*^
ESS^3^, mean (SD)	12.29 (4.65)	3.50 (2.78)	F = 32.718, *p* < .001^*^
Self-reported hours of sleep during night before test, mean (SD)	7.32 (0.14)	7.08 (0.79)	F = 0.371, *p* = .548
Duration NT1 symptoms in years, mean (SD)	15.50 (8.98)	N/A	N/A
Range	3–35		

^1^Body mass index,

^2^Fatigue Severity Scale,

^3^Epworth Sleepiness Scale,

^*^ *p*-value are significant with α ≤ .05.

Participants were asked about sleepiness symptoms during the test day. Seven NT1 participants mentioned sleepiness during the test day. Three of them said that they “possibly” fell asleep during the resting or recovery periods; one participant reported that they “certainly” fell asleep during the last recovery period. Two of these seven participants had a nap right after the experiment. Nine participants with NT1 in total, and six out of the seven NT1 participants with self-reported sleepiness during the test day, used stimulant medications (but not on the test day).

### General outcomes

Differences in the baseline physiological stress markers were not explained by variances in the use of stimulants, number of the test session, or use of oral contraceptives. No baseline differences between the NT1 and control group were observed in the objective physiological stress markers. The NT1 group reported more baseline psychological stress on the STAI-DY1 questionnaire (*p* = .012), but not on the stress component of the VAS questionnaire (*p* = .601).

**Figure 3.**
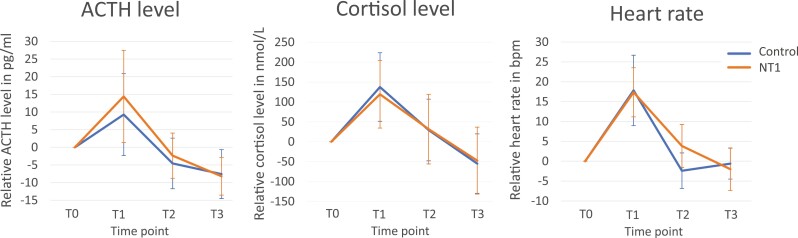
Relative physiological stress measurements over time. ACTH, adrenocorticotropic hormone; NT1, narcolepsy type 1; error bar = 95% CI.

**Figure 4.**
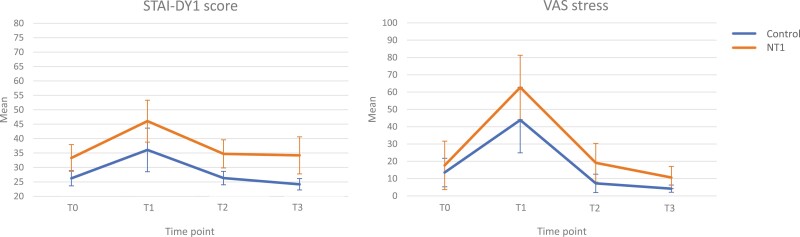
Psychological stress measurements over time. STAI-DY1, the first part of the Dutch version of the State and Train Anxiety Inventory; VAS, visual analog scale; NT1, narcolepsy type 1; error bar, 95% CI.

A clear physiological and psychological stress response was found for the total group between T0 and T1 (immediately after the TSST-G); for ACTH (T0: mean = 20.03, SD = 9.36. vs. T1: mean = 32.78, SD = 18.62; *t* = 2.999, *p* = .006), cortisol (T0: mean = 268.52, SD = 147.15 vs. T1: mean = 396.25, SD = 101.19; *t* = 4.673, *p* < .001), heart rate (T0: mean = 70.77, SD = 10.04 vs. T1: mean = 89.02, SD = 8.05; *t* = 7.747, *p* < .001), STAI-DY1 (T0: mean = 30.04, SD = 7.35 vs. T1: mean = 40.75, SD = 12.88; *t* = 6.586, *p* < .001), and the VAS stress score (T0: mean = 15.73, SD = 19.60 vs. T1: mean = 52.54, SD = 32.60; *t* = 5.318, *p* < .001) (Figures 3 and 4). Figure 3 shows relative stress markers, where the levels are corrected for baseline measurement. Figure 4 shows the absolute levels, without correction for baseline. In these calculations, heart rate measurements during the TSST-G of two participants with NT1 were missing due to technical errors.

For the NT1 group, there was no correlation between the duration of the NT1 symptoms and baseline cortisol level, heart rate, STAI-DY1, or VAS stress outcomes. Baseline ACTH level correlated negatively with the duration of NT1 symptoms (*r* = −.633, *p* = .015), i.e. baseline ACTH levels were lower in those with longer illness duration.

### Physiological stress

Participants with NT1 did not have an altered ACTH (*p* = .820) or heart rate (*p* = .377) after stress compared to controls (Table 2 and Figure 3). Overall, men had a higher ACTH value (mean = 23.57 pg/mL, SD = 12.73) than women (mean = 18.02 pg/mL, SD = 13.24; *t* = 2.720, *p* = .012).

**Table 2.** Physiological Stress Outcome Measurements

	NT1, *n* = 14 Mean (SD)	Controls, *n* = 12 Mean (SD)	Linear mixed model outcome for group differences, *P*-value^1^
ACTH in pg/mL			*t* = 0.231, *p* = .820
T0 (intercept)	20.35 (8.51)	19.64 (10.63)	
T1	34.76 (19.83)	28.93 (15.99)	
T2	17.99 (6.74)	15.07 (7.09)	
T3	12.16 (4.48)	12.06 (5.45)	
Cortisol in nmol/L			*t* = 0.369, *p* = .716
T0 (intercept)	259.37 (134.12)	279.20 (166.50)	
T1	378.55 (101.71)	416.66 (106.38)	
T2	291.01 (72.37)	308.64 (97.34)	
T3	211.86 (52.70)	224.22 (87.92)	
Heart rate in bpm	**NT1, *n* = 12**	**Controls, *n* = 12**	*t* = 0.900, *p* = .377
T0 (intercept)	69.06 (8.86)	72.76 (11.34)	
T1	87.47 (9.13)	90.58 (6.85)	
T2	72.11 (9.44)	70.36 (11.84)	
T3	67.28 (8.13)	72.14 (12.53)	

^1^with the NT1 group as intercept.

**Figure 5.**
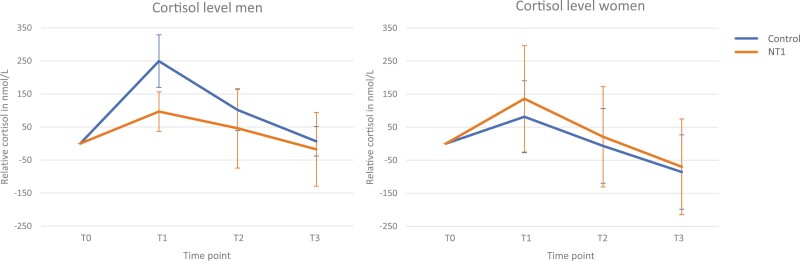
Relative cortisol levels per gender. NT1, narcolepsy type 1; error bar, 95% CI.

For the cortisol levels, the repeated measures LMM analysis did not show a main effect between the groups (*p* = .716; Table 2), but there was an interaction effect of Gender × Group (*p* = .017). A subsequent ANOVA per timepoint and gender showed lower cortisol levels immediately after the TSST-G for men with NT1, than for men from the control group, when cortisol values were corrected for baseline values (*p* = .002; see Figure 5 and Figure S1). Individual plots of cortisol levels over time suggest the opposite trend for women (Figure S2). No differences were found for women with and without NT1, for men on other timepoints, or for the absolute cortisol levels.

Individual plots (Figures S2 and S3) showed that some participants started with high cortisol levels during the baseline assessments (defined as >300 nmol/L). In most participants, the cortisol level immediately after the TSST-G was minimally raised, or even lower than the baseline level. However, high cortisol levels at baseline were not specific for gender or the presence of NT1. Excluding participants with high cortisol baseline levels did not lead to different results in any physiological or psychological stress markers.

The AUCg and AUCi analyses showed no altered patterns in any physiological stress outcome measurements in the total group. In men, the AUCg value for cortisol was not different between groups (*p* = .062).

### Psychological stress

The NT1 group reported more subjective (psychological) stress on the STAI-DY1 (*p* = .002) questionnaire, with higher scores observed at all timepoints. People with NT1 did not report an altered subjective psychological stress, compared to the control group, on the stress component of the VAS questionnaire (*p* = .107) (see Table 3, Figure 4, and Figure S4). Overall, women scored higher on the STAI-DY1 (mean = 35.27, SD = 11.34) than men (mean = 29.33, SD = 8.85; *t* = 2.980, *p* = .007), and there was a trend towards a higher VAS stress score (women: mean = 26.80, SD = 30.27 vs. men: mean = 16.30, SD = 21.43; *t* = 1.755, *p* = .084).

**Table 3.** Psychological Outcome Measurements

	NT1, *n* = 14 Mean (SD)	Controls, *n* = 12 Mean (SD)	Linear mixed model outcome for group differences, *P*-value^1^
STAI-DY 1			t = −3.435, *p* = .002^*^
T0 (intercept)	33.29 (8.03)	26.25 (4.16)	
T1	46.07 (12.5)	36.08 (11.91)	
T2	34.71 (8.45)	26.33 (3.68)	
T3	34.21 (11.15)	24.17 (3.40)	
VAS stress			*t* = −1.635, *p* = .107
T0 (intercept)	17.64 (24.26)	13.50 (12.94)	
T1	62.71 (32.18)	43.83 (29.79)	
T2	19.14 (19.27)	7.25 (8.31)	
T3	10.64 (11.10)	4.17 (3.27)	

^1^with the NT1 group as intercept.

^*^ *p*-value is significant with α ≤ .05.

**Figure 6.**
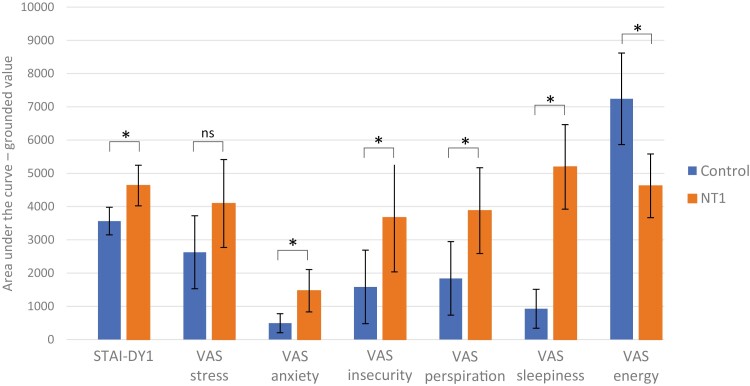
The grounded area under the curve (AUCg) for the STAI-DY1 and VAS questionnaires. STAI-DY1, the first part of the Dutch version of the State and Train Anxiety Inventory; VAS, visual analog scale; NT1, narcolepsy type 1; *, *p*-value are significant with α ≤ 0.05, ns, not significant; error bar, 95% CI.

The higher STAI-DY1 scores in the NT1 group, compared to the control group, were also reflected in the AUCg analysis (F = 9.189, *p* = .006), but not in the AUCi value. The AUCg and AUCi for the VAS stress score were not different between the groups. The NT1 group had higher AUCg levels of self-reported VAS anxiety (F = 8.187 *p* = .009), VAS insecurity (F = 4.908, *p* = .036), VAS perspiration (F = 6.558, *p* = .017), VAS sleepiness (F = 38.884, *p* < .001) and VAS lower energy levels (F = 12.167, *p* = .002) (see Figure 6 and Figure S4). The AUCi analyses only showed an overall increased pattern for VAS anxiety (F = 5.346, *p* = .030).

### Associations between stress markers

The absolute values for all physiological stress measurements positively correlated with each other for the total group (*r* = .394 for ACTH-cortisol; *r* = .348 for ACTH-heart rate; *r* = .248 for cortisol-heart rate; all *p* < .05). The two subjective psychological stress questionnaires also had a strong positive correlation for the total group (*r* = .777 for STAI-DY 1-VAS stress, *p* < .001). The physiological stress markers also positively correlated with the psychological stress markers for the total group (*r* = .239 for ACTH-STAI-DY1; *r* = .366 for cortisol-STAI-DY1; *r* = .362 for ACTH-VAS stress; *r* = .411 for cortisol-VAS stress; *r* = .343 for heart rate-VAS stress; all *p* < .05). Only the correlation between heart rate and STAI-DY1 showed a trend towards significance (*r* = .193; *p* = .053).

## Discussion and Conclusion

We did not find general differences in the objective physiological (endocrine and cardiovascular) stress responses in people with NT1 compared with controls, although men with NT1 had lower cortisol levels immediately after the induced stress than men without NT1. Unexpectedly, we found an increase in overall subjective (psychological) stress in the NT1 group compared to the controls. Additionally, in general, women reported higher psychological stress than men whereas men had overall higher ACTH levels.

The observation of the overall normal physiological stress responses and increased psychological stress responses in people with NT1 is not in line with the decrease in CRH-positive neurons as described earlier [3]. An explanation could be that people with NT1 compensate for the loss of CRH with other hormones, such as vasopressin (AVP). CRH is the main secretagogue for ACTH that is released by the PVN, but its production is also stimulated by AVP. A substantial number of the CRH neurons in the rat PVN co-express AVP in normal conditions [12]. There may be a different role for AVP in the PVN of people with NT1 regarding the secretion of ACTH. Interestingly, the remaining CRH cells in the postmortem PVN of people with NT1 were mostly AVP-positive and the percentage of CRH cells that co-expressed AVP was increased by 234% [32]. Earlier studies showed that chronic stress alters the expression of AVP and CRH in the hypothalamus. Prolonged psychological stress in rodents increased AVP synthesis in CRH-producing PVN neurons while CRH remained unchanged or decreased. In those rodents, the proportion of hypothalamic CRH neurons that co-expressed AVP was also increased [12]. This suggests a shift in the AVP/CRH signaling in people with NT1 after prolonged psychological stress. In this case, decreased ACTH levels may partially be avoided due to increased AVP synthesis in the PVN compensating for the reduced CRH-positive cells. This may be why we found generally elevated psychological stress and normal ACTH levels in NT1.

Because ACTH correlated negatively with NT1 disease duration, AVP may not fully compensate for the lack of CRH release in the longer term. It is also possible that the loss of CRH is a consequence—or collateral aspect—of NT1 rather than involved in the development of NT1. Additionally, the decrease of CRH-positive neurons as found earlier in people with NT1 was observed in postmortem tissue [3]. How much CRH production is reduced in the living NT1 population or in our NT1 study participants is unknown. We cannot be sure that the hypothesized loss of CRH neurons in NT1 can explain our results.

Another factor in the overall unaltered physiological stress response in people with NT1 could be a compensation of reduced CRH expression by a stronger stress experience in the NT1 group. The HPA response in people with NT1 may be lower than controls with the same amount of stress. However, the NT1 group in our experiment reported generally more psychological stress than the control group. This suggests that the remaining CRH neurons in the PVN of people with NT1 might be more activated, due to higher stress appraisal, resulting in similar ACTH and cortisol levels as in controls. Earlier research showed that activation of CRH neurons in the mouse PVN are more activated immediately after stress exposure and this neural activity dropped rapidly after the presentation of appetitive stimuli [33].

There are electrophysiological interactions between Hcrt and CRH in the rodent lateral hypothalamus [8]. CRH axon terminals make direct contact with Hcrt cells and CRH-R1 and -R2 receptors are expressed by Hcrt cells. The administration of CRH depolarizes Hcrt cells through these CRH receptors [34]. In addition, PVN neurons depolarize after the application of Hcrt neuropeptides [35]. CRH is found in many brain regions, such as in the bed nucleus of the stria terminalis and the amygdala, but CRH in the PVN plays a crucial role in activating the stress response [9]. Because specific loss or silencing of CRH in the PVN results in altered HPA functioning, it is unlikely that CRH in other regions can compensate for the loss of CRH in the PVN by generating a stress response [9, 15, 16]. Hcrt does not affect basal ACTH release in rats, but can inhibit CRH-stimulated ACTH secretion in vitro [36]. The absence of CRH- and Hcrt-producing neurons in NT1 might even out the effects on ACTH secretion and the further stress response in this population—with AVP as the main hypothalamic effector in the HPA-axis.

Interestingly, the expected decrease in HPA response was observed in the cortisol levels in men with NT1 compared with control men immediately after stress exposure. A decrease was undetected at any other timepoint in men; nor in the ACTH stress response; it was also not detected in women. Our results may suggest an opposite physiological stress response for men and women with NT1. However, the sample size was too small to draw reliable conclusions regarding gender differences. The postmortem brain tissue used in a previous publication, which showed reduced CRH-positive neurons in people with NT1, was predominantly obtained from women (four women, one man) [3]. This contrasts with the direction of the findings of the current study. The ACTH and cortisol results are not statistically explained by other factors such as high baseline cortisol levels in some of the participants, a different TSST-G session, use of medication, use of oral contraception, self-reported sleep duration, or the time the participant got out of bed on the test day. However, the role of medication cannot be ruled out since there was high variation in the use of medications between participants (e.g. dosage, time of intake, and duration of treatment) that we were not able to account for.

A limitation of the current study is that the sample size is too small to draw firm conclusions on any gender differences. Also, controls in this study are matched for age up to six years difference with a participant in the NT1 group. A more restricted range for the age-matched pairs will even limit the potential effect of age on the outcome measurements.

Moreover, cortisol in the cerebrospinal fluid (CSF) generally correlates with cortisol levels in the periphery [37–40], but central ACTH levels do not necessarily correspond to peripheral ACTH levels. Acute manipulations of the HPA-axis (by administration of stimulating or inhibiting agents) in rhesus monkeys mainly influenced peripheral ACTH levels rather than ACTH in the CSF [41]. Furthermore, people with anorexia nervosa showed normal ACTH plasma levels while ACTH levels in the CSF were reduced [38]. Therefore, the plasma ACTH measurements in this study may miss potential dysregulations of ACTH secretion within the central nervous system. We did also not include AVP measurements. Future research on the activity of AVP in the PVN in people with NT1 may explain why the elevated psychological stress in NT1 in our study is not reflected in the physiological stress markers. Furthermore, information regarding phenotype severity and more details about medication use will be valuable additions to the measurements since this can vary a lot between people with NT1. Some of the participants with NT1 who are treated with stimulants reported sleepiness during the test day in which they did not take their stimulant medication. Sleepiness or long-term effects of stimulant use in these patients may have altered the results. Even though no statistical differences were shown between stimulant users and non-stimulant users, alterations cannot be ruled out.

Lastly, the subjective (psychological) stress results should be interpreted carefully. People with NT1 reported an altered stress level measured by the STAI-DY1 questionnaire, but not when measured by the stress component of the VAS questionnaire. Despite the outcomes of the STAI-DY1 and VAS stress questionnaires having a strong positive correlation, (one of) the questionnaires may not be a completely accurate measure of psychological stress. It remains unclear whether the loss of CRH production is involved in the symptomatology experienced by people with NT1. Understanding the underlying mechanisms is important to predict how patients respond to newly developed treatments, such as the Hcrt receptor 2 agonists currently being tested in clinical trials.

Despite an overall elevated psychological stress level in people with NT1, the physiological stress response was not altered. The objective physiological stress measurements were the most direct targets of CRH and thus considered our most critical outcomes. We conclude that people with NT1 have a normal reaction to acute stress compared to people without NT1, apart from men with NT1, who may have a lower cortisol stress response than control men. Additional research is needed to determine gender differences in the stress response of people with NT1 and the relationship between CRH-producing neurons and the HPA-axis in NT1.

## Supplementary material

Supplementary material is available at *SLEEP* online.

zsae265_suppl_Supplementary_Material

## Data Availability

The data underlying this article will be shared on reasonable request to the corresponding author.
