# Supplementary material for: Physiological and psychological stress reactivity in narcolepsy type 1
Source: Sleep. 2024 Nov 15;48(3):zsae265. doi: 10.1093/sleep/zsae265 (PMC11893539; doi:10.1093/sleep/zsae265)
Supplement: zsae265_suppl_Supplementary_Material [file zsae265_suppl_supplementary_material.docx]

# **Physiological and Psychological Stress Reactivity in Narcolepsy Type 1**

Marieke Vringer^1,2^, Denise Bijlenga^1,2^, Jingru Zhou^1,2^, Onno C. Meijer^3^, Christiaan H. Vinkers^4,5,6,7^, Gert Jan Lammers^1,2^, Rolf Fronczek^1,2^

1. Stichting Epilepsie Instellingen Nederland (SEIN), Sleep-Wake Center, Heemstede, Netherlands
2. Leiden University Medical Center (LUMC), Department of Neurology, Leiden, Netherlands
3. Leiden University Medical Center (LUMC), Department of Neuroendocrinology, Leiden, Netherlands
4. Amsterdam University Medical Center (AUMC), Department of Psychiatry and Department of Anatomy & Neurosciences, Amsterdam, Netherlands
5. Amsterdam Neuroscience, Mood, Anxiety, Psychosis, Stress & Sleep program, Amsterdam, Netherlands
6. Amsterdam Public Health, Mental Health program, Amsterdam, Netherlands
7. GGZ InGeest, Academic Working Place Depression, Amsterdam, Netherlands

Corresponding author:

Rolf Fronczek

Sleep-Wake Center SEIN

Achterweg 3, 2103 SW Heemstede

r.fronczek@lumc.nl

**Table S1. Overview of medication use per participant.**

|  | **Participant** | **Medication** | **Indication** |
| --- | --- | --- | --- |
| **Control**  **group** | 1 | - |  |
|  | 2 | - |  |
|  | 3 | - |  |
|  | 4 | - |  |
|  | 5 | Homeopathic remedies | Menopausal symptoms |
|  | 6 | Levothyroxine | Thyroid dysfunction |
|  | 7 | - |  |
|  | 8 | - |  |
|  | 9 | - |  |
|  | 10 | Naproxen | Pain with hypermobility |
|  | 11 | - |  |
|  | 12 | Betamethasone ointment (not on daily basis, not on few days before and not on testing day) | Eczema |
| **NT1 group** | 13 | Pitolisant | NT1 |
|  | 14 | - |  |
|  | 15 | Sodium oxybate  Pitolisant  Methylphenidate (not on testing day) | NT1  NT1  NT1 |
|  | 16 | Pitolisant  Methylphenidate (not on daily basis, not on testing day) | NT1  NT1 |
|  | 17 | Modafinil (not on testing day) | NT1 |
|  | 18 | Methylphenidate (not on testing day)  Fluticason (nasal spray, not on testing day) | NT1  Nasal mucosa |
|  | 19 | Sodium oxybate  Pitolisant  Esomeprazol (not on daily basis)  Sumatriptan | NT1  NT1  Stomach complains  Migraine |
|  | 20 | Modafinil (not on testing day)  Sodium oxybate  Cetomacrogol (3x per day, not on testing day) | NT1  NT1  Eczema |
|  | 21 | Methylphenidate (not on testing day)  Sodium oxybate | NT1  NT1 |
|  | 22 | Methylphenidate (not on testing day)  Clomipramine (low dose)  Pitolisant  Sodium oxybate  Levothyroxine | NT1  NT1 (cataplexy)  NT1  NT1  Thyroid dysfunction |
|  | 23 | Methylphenidate (not on testing day)  Sodium oxybate | NT1  NT1 |
|  | 24 | Sodium oxybate  Paroxetin (low dose) | NT1  NT1 (cataplexy) |
|  | 25 | Sodium oxybate  Pitolisant  Modafinil (not on testing day) | NT1  NT1  NT1 |
|  | 26 | Sodium oxybate  Modafinil (not on testing day) | NT1  NT1 |

NT1 = Narcolepsy type 1.


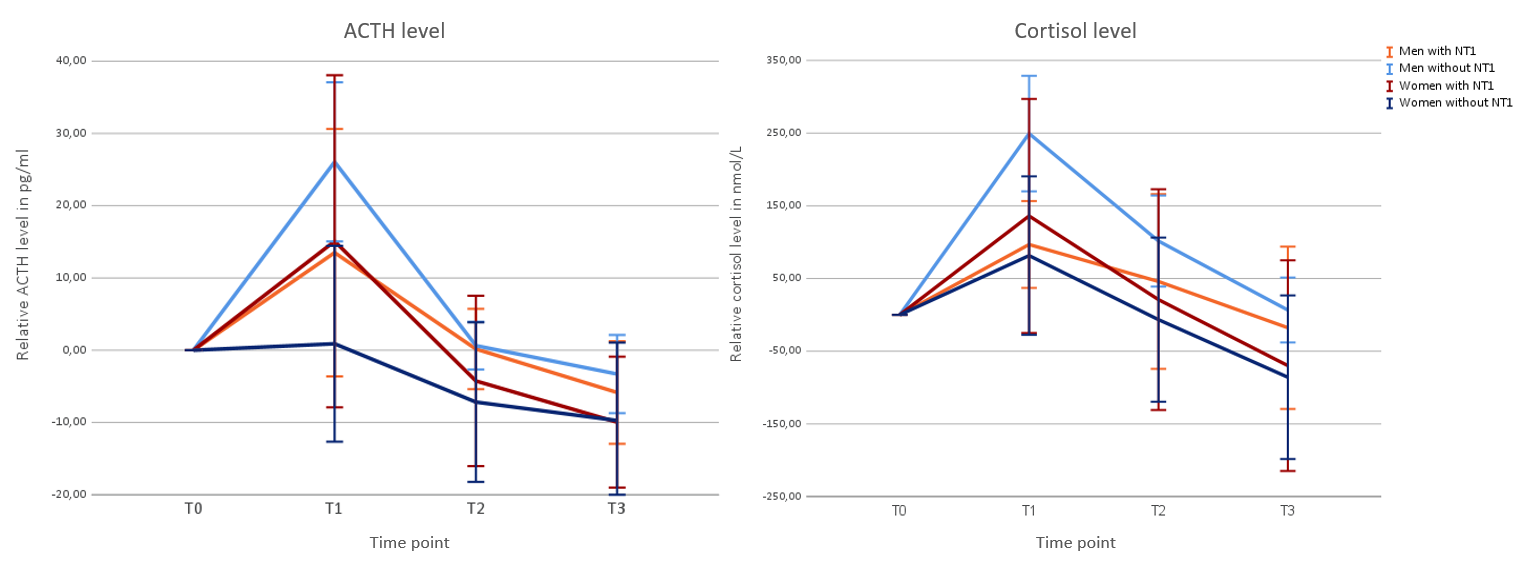


**Figure** **S1. Relative ACTH and cortisol levels per category.** ACTH = adrenocorticotropic hormone, NT1 = narcolepsy type 1, error bar = 95% CI


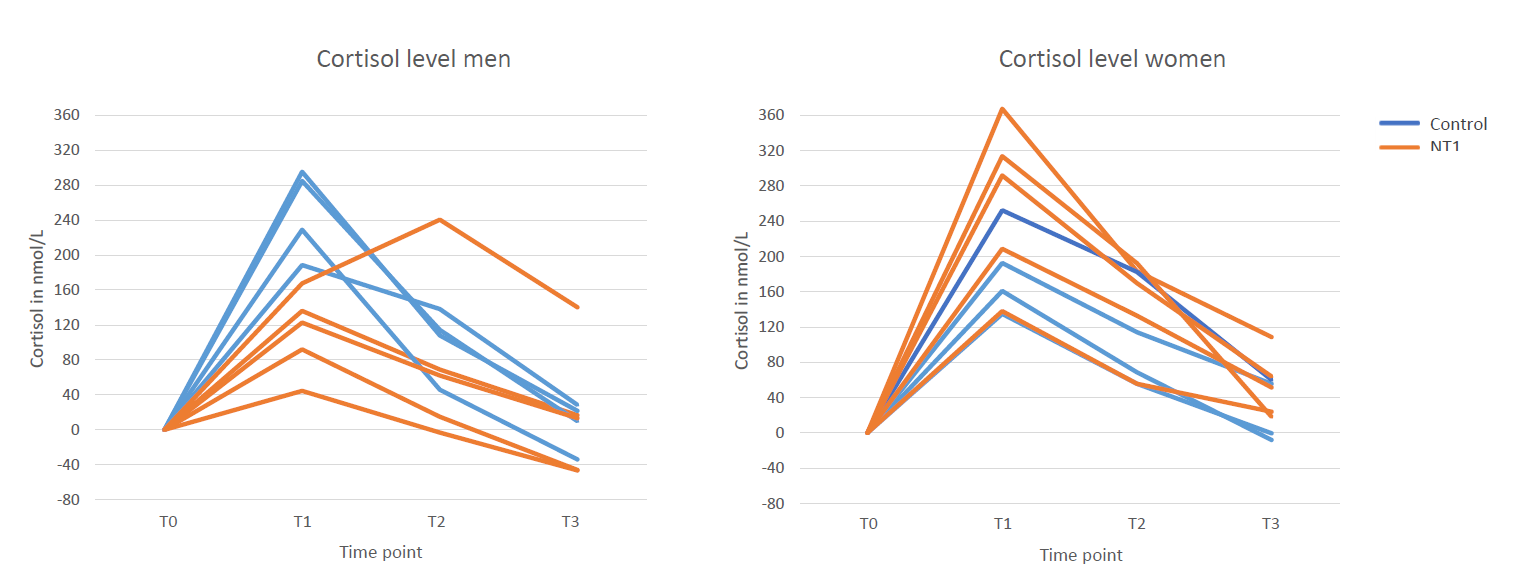


**Figure S2. Relative cortisol levels per participant with normal baseline cortisol level.** NT1 = narcolepsy type 1.


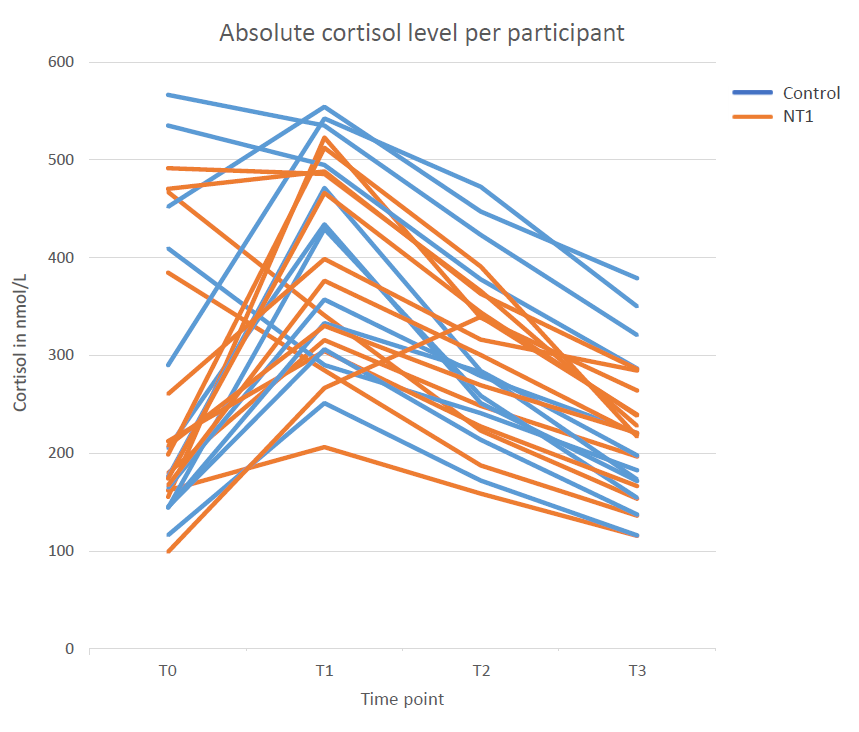


**Figure S3. Absolute cortisol level per participant.** NT1 = narcolepsy type 1.


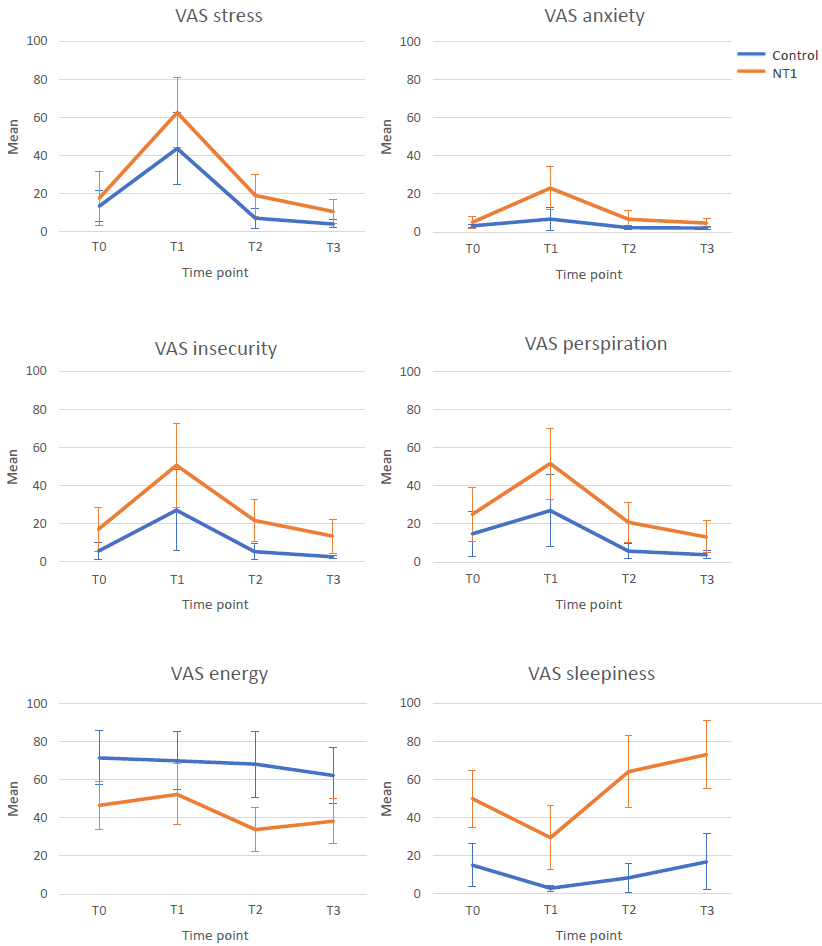


**Figure S4. Absolute VAS outcomes over time.** VAS = visual analog scale, NT1 = narcolepsy type 1, error bar = 95% CI
